# Special Issue from the 2017 International Conference on Mathematical Neuroscience

**DOI:** 10.1186/s13408-018-0069-5

**Published:** 2019-01-07

**Authors:** Zachary P. Kilpatrick, Julijana Gjorgjieva, Robert Rosenbaum

**Affiliations:** 1 Department of Applied Mathematics, University of Colorado Boulder, Boulder, USA; 2 Computation in Neural Circuits Group, Max Planck Institute for Brain Research, Frankfurt, Germany; 3 Department of Applied and Computational Mathematics and Statistics, University of Notre Dame, Notre Dame, USA

## Abstract

The ongoing acquisition of large and multifaceted data sets in neuroscience requires new mathematical tools for quantitatively grounding these experimental findings. Since 2015, the International Conference on Mathematical Neuroscience (ICMNS) has provided a forum for researchers to discuss current mathematical innovations emerging in neuroscience. This special issue assembles current research and tutorials that were presented at the 2017 ICMNS held in Boulder, Colorado from May 30 to June 2. Topics discussed at the meeting include correlation analysis of network activity, information theory for plastic synapses, combinatorics for attractor neural networks, and novel data assimilation methods for neuroscience—all of which are represented in this special issue.

## Introduction

There is a growing number of large international collaborations between experimentalists and theorists addressing pressing problems in neuroscience [1, 2]. The International Conference on Mathematical Neuroscience (ICMNS) contributes to this effort by training young researchers in modern tools of mathematical neuroscience, providing a forum for new research developments, and promoting discussion about current problems. Ultimately, we hope ICMNS will foster new research collaborations by focusing on mathematical questions and techniques emerging from studying open problems in neuroscience. Since its inception, the conference has featured talks that span the brain’s spatiotemporal scales: from the stochastic dynamics of subcellular mechanisms to the complex spatiotemporal patterns of large-scale neuronal networks, and from submillisecond spiking to learning spanning years. Mathematical theories underlying this work include ideas from mean field theory, stochastic processes, spatiotemporal dynamics, network and graph theory, statistical mechanics, and higher order statistics.

**Figure 1:**
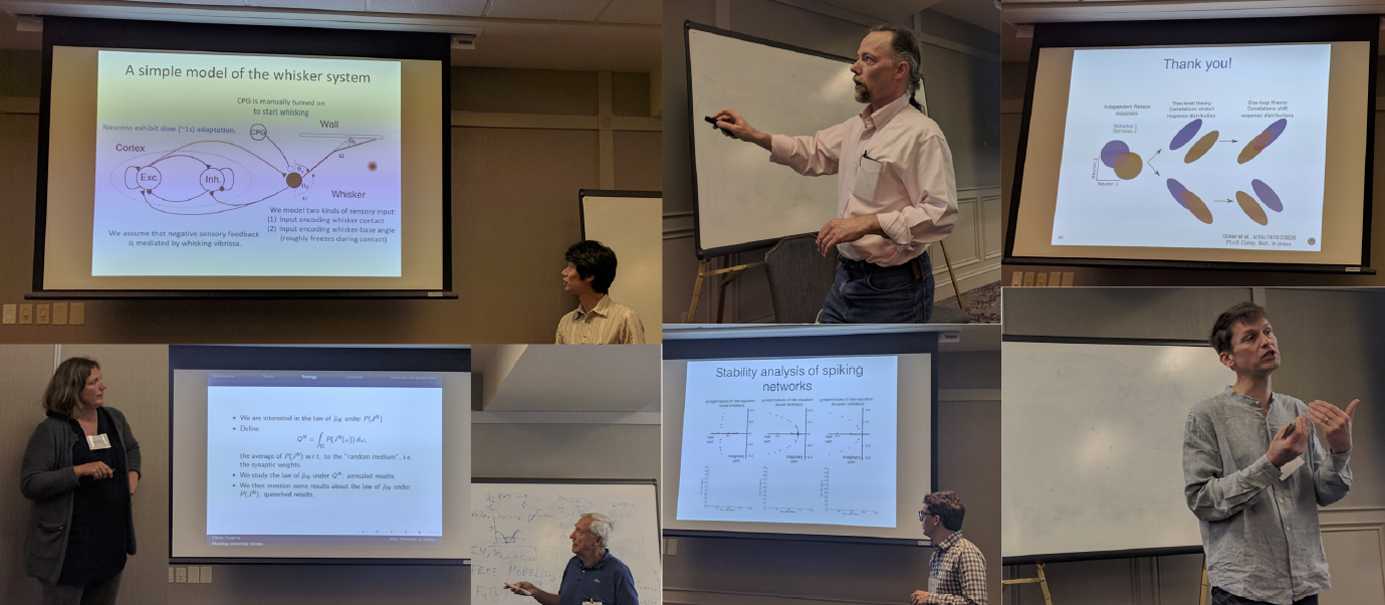
Selected tutorial, plenary, and parallel presentations from ICMNS 2017. Top row, left to right: Taro Toyoizumi (RIKEN Institute for Brain Science) presenting a theory of neural gain modulation by closed-loop environmental feedback; Peter Thomas (Case Western Reserve University), defining the phase of a stochastic oscillator; Brent Doiron’s (University of Pittsburgh) tutorial on neural variability in networks. Bottom row: Sophie Deneve (École normale supérieure, Paris) speaking about efficient coding in spiking networks; Olivier Faugeras (INRIA - Sophia Antipolis) discussing correlations in thermodynamic limits; Robert Rosenbaum (University of Notre Dame) on spatiotemporal dynamics in spiking neural network models; Nicolas Brunel (Duke University) on minimal biophysical models of synaptic plasticity

The first (2015) and second (2016) ICMNS were held in Juan-les-Pins, France, after which the meeting was held in Boulder, Colorado (2017)—the focus of this special issue.^1^ Building on the structure of previous meetings, the conference began with a tutorial day followed by a three-day-long main meeting. The tutorial day was organized to attract and train young researchers on current methods in mathematical neuroscience. There were two tracks, providing a broad swath of topics (see Fig. 1), including balanced networks, information theory and geometry, efficient coding in spiking networks, plasticity, and stochastic hybrid systems. In line with these efforts, we present two tutorial reviews, one on stochastic hybrid methods [3] and the other on data assimilation methods in neuron models [4].

The main meeting featured three days of plenary speakers, parallel sessions, and poster presentations (see Fig. 1), sampled this special issue’s research articles [5–9]. Presentations at ICMNS focus on mathematical methods and models developed to study open problems in neuroscience. This is distinct from presentations at other computational neuroscience meetings (e.g., CoSyNe, CNS, and NeurIPS), which emphasize new neuroscience or computational methods, with less focus on mathematics and tractable models. Also, in contrast to other applied mathematics meetings focused on mathematical modeling (e.g., SIAM Applied Dynamical Systems and SIAM Life Sciences), most individuals at ICMNS have a basic background in neuroscience. Therefore, ICMNS has a unique advantage in that it can focus more deeply on new mathematics emerging in neuroscience. We discuss examples of this trend, published in this special issue.

## Tutorial reviews

The two tutorials demonstrated the importance of considering uncertainty and variability in the brain and in the data collection process required to fit models of neural dynamics. Neuronal spiking [10], as well as the state dynamics of underlying ion channels and receptors [11], can be highly stochastic. To understand how stochasticity emerges at the macroscopic level, it is important to scale up microscopic models of such fluctuations. Bressloff and MacLaurin (2018) review stochastic hybrid methods, which allow for the detailed analysis of partially deterministic Markov processes (PDMPs) that emerge from models in cellular neuroscience [3]. Considering variability and uncertainty is also important when fitting parameterized models to data. Along these lines, Moye and Diekman (2018) review data assimilation methods as applied to fitting parameterized conductance-based models [4]. They also include MATLAB code for implementing these methods as supplementary material.

Fluctuations at the subcellular level can shape the response properties of single neurons and circuits [12–14]. Ion channels open and close and can be modeled by a continuous time Markov process, where the finite number of channels causes spontaneous spiking in conductance-based models due to channel fluctuations [12, 15]. Bressloff and MacLaurin (2018) review the mathematical framework needed to analyze such stochastic systems, leveraging their piecewise deterministic nature [3]. Stochastic transitions occur at discrete time, but in between the dynamics evolves deterministically. This is captured by differential Chapman–Kolmogorov (CK) equations, which can be reduced by quasi-steady-state (QSS) approximations to derive lower order corrections to mean field equations which are valid in the infinite system size limit. Moreover, quasipotentials can be derived using the Wentzel–Kramers–Brillouin (WKB) approximation to compute first passage time statistics in metastable systems [12]. Such metastability can emerge in stochastic hybrid systems which in the mean field limit become conductance-based neuron models. The stochastic evolution of action potentials in such models can be characterized by analyzing the corresponding CK equations describing the population evolution of ion channel states [3]. The authors also discuss models with stochastic gap junctions and diffusion in randomly switching environments [16], as well as models of intracellular transport in axons and dendrites [17]. They conclude their tutorial by analyzing the phase dynamics of oscillators that evolve according to PDMPs using a variational approach, which more precisely characterizes phase response properties and the time to escape the neighborhood of a stochastic limit cycle. The authors suggest these methods could be scaled up to more complex biophysical processes or even synaptically coupled networks. For example, the individual spikes of a neural population might be treated as the discrete stochastic process, and the synaptic dynamics as the intervening deterministic process.

In a complementary tutorial review, Moye and Diekman (2018) discuss data assimilation as applied, for example, to fit conductance-based models to current-clamp data [4]. In general, data assimilation uses direct observations to improve estimates of a model of some corresponding system, distinguishing it from other forms of statistical learning which do not use dynamical models. To estimate the system state of interest over time, a cost function is typically defined and minimized to define the particular data assimilation method: Variational methods (weak 4D-Var) seek minima by formulating an adjoint problem [18]. Sequential data assimilation uses information from previous time points and the current observation to estimate the state. These are known as filters, the Kalman filter for example [18], predicting state estimates with a model and correcting them with observations. Moye and Diekman (2018) discuss unscented Kalman filters (UKFs), which approximate the dynamics of nonlinear systems by calculating the sample means and variances of the nonlinearly transformed state variables [19]. Importantly, the nonlinear transformation remains in the updating process, but the resulting prediction is still defined by Gaussian statistics. Considering nonlinearities is especially important to estimate nonlinear features like bifurcations. The authors apply the UKF to the Morris–Lecar model near a Hopf bifurcation, saddle-node on a limit cycle, and a homoclinic. Parameters, voltage values, and bifurcation points are well identified using the UKF, which would be a challenge for linear filtering methods. The weak 4D-Var method performs worse, since it can settle into local minimizers of the cost function, far from global minima, especially in metastable parameter regimes. An analysis of the Morris–Lecar model in bursting regimes shows the UKF performs well at estimating parameters and states in some bursting regimes. Elliptic square wave bursters can be harder to fit, but improvements may be possible by treating the slow and fast dynamics as separate state estimation problems [20]. Data assimilation will continue to be an important set of methods as more neural data comes online that must be combined with nonlinear models. The statistical approach by Moye and Diekman (2018) may be particularly exciting for those focused on dynamical systems in mathematical neuroscience, as it can preserve an account for the inherent nonlinearities of neural systems [4].

The tutorials thus provide complementary analyses and techniques for addressing stochasticity in neural systems. Bressloff and MacLaurin (2018) analyze mechanistic models accounting for stochasticity at the microscopic level and discuss ways of scaling these models up to more macroscopic quantities [3]. Alternatively, Moye and Diekmann (2018) suggest means of dealing with such stochasticity as a statistical problem [4], so noisy data can be filtered properly to reliably fit deterministic models—these can be the mean field counterparts of stochastic neuronal models.

## Research articles

The research articles in this special issue analyze models of neural systems at multiple scales. Intracellular calcium signals are important for synapse-to-nucleus communication, and can take the form of waves which Breit and Queisser (2018) study in a reaction-diffusion model of calcium propagation [7]. At the neuron level, Mokhtari et al. (2018) study how short-term plasticity impacts neural information propagation across synapses, exploring the effect of muscarine treatment on hippocampal neurons [6]. At the microcircuit level, Ferrario et al. (2018) analyze bifurcations that determine transitions between swimming and synchrony modes in a simple model of the *Xenopus* tadpole spinal central pattern generator (CPG) [8]. Moving to large-scale networks, Barreiro and Ly (2018) derive relationships between pairwise spike count correlations and firing rates in heterogeneous networks, extending linear response theory [5]. Lastly, Hedrick and Zhang (2018) use local stability analysis to study the operational modes of an attractor neural network model of spatial working memory, showing how output patterns depend on conflicting external inputs [9]. These studies demonstrate the wide array of mathematical techniques being developed to understand the dynamics and coding processes of neural systems.

The propagation of intracellular calcium is vital to signal transduction, synaptic transmission, and the dynamics of action potentials [21]. Calcium-induced calcium release brought about via ryanodine receptor (RyR) channels on the endoplasmic reticulum (ER) promotes such calcium propagation [22]. Breit and Queisser (2018) specifically study how dendritic geometry and the ER interact to influence calcium wave dynamics [7], modeling the ER within the dendrite as a cable sheathed by another cable layer. Interestingly, they find that a minimal RyR density, which scales inversely with ER surface area, is needed to ensure conditions for traveling wave propagation. Along these lines, as the RyR density increases, so does the speed of calcium waves. In future studies, the authors plan to explore systems with heterogeneous RyR distributions and propose that wave propagation failure could be analyzed using averaging and homogenization [23]. Alternatively, the authors propose to study the asymptotic limit of thin dendrites, so the model could be treated as one-dimensional.

Building on this theme of the role of calcium in neural processing, Mokhtari et al. (2018) investigate the information processing properties of a dynamical model of a synapse subject to short-term synaptic plasticity based on calcium-dependent modulation of vesicle release and recovery probabilities [6]. As the model has been validated by data from hippocampal GABAergic synapses [24], it provides a testbed for the effects of muscarine application, which inhibits presynaptic calcium channels, on information transfer in the synapse. By simplifying the presynaptic calcium kinetics, the authors derive a nonlinear map for the calcium concentration, the probability of vesicle release, and the concentration of vesicles in the releasable pool. These vesicle dynamics promote short-term depression which acts as a frequency-dependent filter on Poisson spike trains, as shown in previous work using linear response techniques [25, 26]. Mokhtari et al. (2018) extend these previous findings by analyzing coding using mutual information measures and observe that muscarine application reduces information transfer across input frequencies. The effects of muscarine are modeled by reducing interspike calcium influx to reflect the fact that muscarine binds to acetylcholine receptors and inhibits presynaptic calcium channels. In future work, the authors propose to consider stochastic binding processes using continuous time Markov chain models. In addition, the simple release probability model could be embedded into microcircuit models to understand how short-term plasticity shapes oscillations and rhythms.

Ferrario et al. (2018) study oscillations and synchrony in the context of a reduced model of the *Xenopus* tadpole central pattern generator (CPG) [8]. CPGs serve an important purpose in motile organisms by autonomously generating rhythmic activity without periodic forcing. The *Xenopus* CPG contains multiple cells whose natural rhythmic pattern shifts from an anti-phase oscillation, ideal for swimming, to an in-phase oscillation, ideal for limb movement, as it undergoes metamorphosis [27]. To analyze this transition, the authors consider a reduced two-unit model of the CPG circuit. Each unit is comprised of an excitatory and an inhibitory Hodgkin–Huxley cell. Coupling within units is dominated by slow NMDA producing recurrent excitation, and coupling between units is dominated by glycinergic synapses from inhibitory to excitatory cells. By varying the strength of synaptic coupling, the system switches between anti-phase and in-phase (swimming vs. synchrony) oscillation regimes. These bifurcations can be revealed via numerical continuation techniques that identify the Neimark–Sacker and period doubling bifurcations that bound multistable regimes. When both behaviors are present via metastability, different initial conditions can unmask either oscillatory behavior, and transitions are possible via stochastic perturbations to the voltage equations and gating variables. The reflection symmetry of the system can be exploited to simplify the bifurcation analysis, although the results are robust to mild symmetry breaking. Overall, their analysis provides intuition for development-based transitions in rhythmic behavior using a simplified model with an accessible bifurcation structure.

The remaining articles focus on analyzing models of large networks, which can exhibit intricate relationships between network architecture and the resulting spatiotemporal dynamics. Spike count correlations generalize the notion of spike train synchrony to longer timescales. Barreiro and Ly (2018) study how the correlation between two neurons’ spike counts is co-modulated with their firing rates in a wide range of parameter regimes [5]. The nonlinearity of single neuron dynamics intertwines correlations and firing rates in a way that depends sensitively on the neurons’ dynamical state, and therefore depends on the parameters that control single neuron dynamics, as well as the neurons’ input statistics. They use a fundamental relationship derived from linear response theory in which the correlation between two neurons’ spike counts is proportional to the correlation between their synaptic input currents multiplied by the geometric mean of their susceptibility functions, which measure how sensitively the neurons respond to input current modulations. This relationship allows the correlation-rate relationship to be studied using Fokker–Planck equations for the evolution of the neurons’ membrane potential densities. While previous work used the same approach to conclude that correlations can increase with firing rate [28], Barreiro and Ly (2018) find that the correlation-rate relationship is richer than previously thought and depends sensitively on which parameters are modulated and how. These findings have implications for neural population coding, which depends on both rates and correlations.

The dynamics of large-scale neuronal network dynamics can also be accounted for by spatially-organized neural field models. These models can be amenable to local and weakly nonlinear stability analysis, especially when the network connectivity exhibits symmetries or is near-symmetric [29]. Hedrick and Zhang (2018) analyze a model of the hippocampal network underlying spatial navigation, in which individual cells can possess multiple place fields [9]. Such multiplicities in place fields have been observed in recordings from rat hippocampus, as the animal navigates large environments [30]. To study the emergence of such multiplicities and their effect on model network dynamics, the authors determine the stability of model equilibria as the spatial domain size is increased. In small environments, when two conflicting locations are presented as inputs to the model, the network operates in a winner-take-all (WTA) regime, so a single input location is represented by a single bump of activity. As the scale of the environment is increased, there is a critical size beyond which two conflicting inputs can be represented by two distinct bumps. This phase transition is characterized through numerical simulations and a local stability analysis which reduces the neural field model to a two-unit model tracking the heights of the two possible bumps. Phase plane and bifurcation analysis can then be used to identify the boundaries of the WTA and combinatorial modes, as well as hysteresis. The authors also show how such analyses could be extended to multiple inputs, relevant to perceptual and decision-making models [31].

The modeling and analysis in these articles link cellular and network properties, and observed neural activity patterns and behavior, and also advance new mathematical techniques for analyzing models and data in neural systems.

## Conclusions and outlook

ICMNS 2017 was attended by over 140 researchers from the mathematical neuroscience community. The plenary talks and contributed presentations demonstrated a close connection between new mathematical methods and insights in neuroscience. To cope with the growth in data collection, often involving a combination of neural and behavioral recordings, sophisticated and grounded mathematical models are important. However, these models should be accessible to some form of analysis so that low-dimensional relations between system parameters and behavior can be inferred. This was an important goal of most presentations at ICMNS 2017.

Moreover, the meeting continues to create an open environment for young researchers in mathematical neuroscience. The tutorial day, which provides an introduction and training to new mathematical methods in neuroscience, is an important part of the meeting structure. With NSF funding, we were able to provide travel funding to ten students to attend the conference, most of whom were members of underrepresented groups in STEM. Furthermore, of the ten plenary speakers, four were women, which earned us a well above average rating by biasneurowatch for gender diversity.^2^ Also, close to 50% of the accepted contributed presentations were by women. Thus, we think this was a considerable success for gender diversity at neuroscience conferences.

We hope that this momentum will continue in future instantiations of ICMNS. In 2018, the conference was held again in Juan-les-Pins, France, with a similar program including a day of tutorials and a three-day main meeting. In 2019, ICMNS will be held in Copenhagen, Denmark from June 23 to 26. In the years to come, we hope the scope of the conference will expand further to draw in both more participants from the mathematical sciences and more neuroscientists with an interest in leveraging new mathematical tools in their work.

## Footnotes

1 See http://www.math.utah.edu/~kilpatri/icmns_program_final.pdf for the conference program.

2 ICMNS 2017 was more than two standard deviations above the base fraction of female plenary speakers at neuroscience meetings: https://biaswatchneuro.com/category/5-more-than-2std-above-base-rate/
